# Treatment non-adherence among methadone maintenance patients and associated factors: a multicenter, cross-sectional study in Vietnam

**DOI:** 10.1186/s12954-024-01040-8

**Published:** 2024-07-03

**Authors:** Huong Thi Thanh Nguyen, Dai Xuan Dinh

**Affiliations:** Faculty of Pharmaceutical Management and Economics, Hanoi University of Pharmacy, 13-15 Le Thanh Tong, Hoan Kiem District, Hanoi City, 111000 Vietnam

**Keywords:** Adherence, Associated factor, Compliance, Methadone maintenance treatment, Non-adherence, Social support

## Abstract

**Objective:**

This multicenter, cross-sectional study was conducted to investigate the prevalence of treatment non-adherence and its associated factors among methadone maintenance patients in Vietnam.

**Methods:**

This secondary data analysis was conducted using the data from a previous study. Six hundred patients were interviewed face-to-face to collect data on their demographic characteristics and social support. Information about the treatment characteristics and patients’ non-adherence was gathered from medical records and books monitoring their treatment process. Treatment non-adherence was defined as missing at least one methadone dose in the last three months.

**Results:**

The overall prevalence of non-adherence was 45.7%. The average social support score of patients who completely adhered to treatment was significantly higher than that of those who did not (*p* < 0.001). In the multivariate logistic regression model, for each one-unit increase in social support (one score), treatment time (a year), and patient’s monthly income (one million Vietnam dongs), the odds of non-adherence decreased by 28% (aOR = 0.72, 95%CI 0.59–0.88, *p* = 0.002), 15% (aOR = 0.85, 95%CI 0.80–0.91, *p* < 0.001) and 9% (aOR = 0.91, 95%CI 0.85–0.97, *p* = 0.004), respectively. Patients living in Son La (a mountainous province) were 1.72 times (95%CI 1.09–2.71) more likely to be non-adherent as compared to those in other areas (*p* = 0.020). As per univariate analyses, other associated factors could be age, education level, family monthly income, occupation, and opioid relapse (*p* < 0.001).

**Conclusions:**

A high non-adherence rate was found among Vietnamese methadone maintenance patients. Interventions involving social support, occupation, income, and education are needed to improve their treatment adherence.

## Introduction

Globally, one of the severe public health problems is drug use, which can have detrimental effects on users’ health. Recently, the increasing ubiquity of the Internet and social networks has facilitated online sales and users’ access to drugs. As per the statistics of the United Nations Office on Drugs and Crime, approximately 284 million people aged 15–64 worldwide used drugs at least once in 2020, including 38.6 million cases suffering from drug use disorders. Cannabis (209 million users), opioids for non-medical purposes (61 million), amphetamine-type stimulants (ATS) (34 million), and cocaine (21.5 million) were four types of drugs commonly used. In 2019, about 494,000 deaths and 30.9 million years of healthy life lost were attributed to drug use [1, 2]. In Vietnam, as of December 2020, there were roughly 235,000 people using drugs (ATS users: 70–80%) [3].

Methadone maintenance treatment (MMT), a long-term therapy, can engender many beneficial effects on patients’ health. MMT programs have been launched in Vietnam since 2008. At present, besides the subsidization from the government, a patient must pay roughly 0.5US$ per day for MMT. Before the year 2021, patients must go to clinics daily, receive methadone, and take this medicine under the strict supervision of health staff [4]. In three provinces, including Dien Bien, Lai Chau, and Hai Phong, multiday take-home methadone programs have been piloted since April 2021. As of December 2022, among 51,000 MMT patients in Vietnam, about 3000 patients received multiple take-home methadone doses instead of having to visit clinics daily [5].

Take-home methadone programs have been implemented for the sake of convenience for patients and reinforced their therapy attendance. Treatment adherence is the most essential requirement for being enrolled in these programs. MMT patients have to demonstrate consistent compliance with the medication treatment under the supervision of medical staff. The success of MMT significantly relies on treatment adherence, a factor associated with reduced risk of dropout among patients. However, the high prevalence of non-adherence and dropout among MMT patients was witnessed in many previous studies: 73.4% in Yunnan, China [6], 52.2% in Xi’an, China [7], 62% in Guangzhou, China [8], 47.5% in Canada [9], 56.7% in Nam Dinh, Vietnam [10], and 38.2% in Ho Chi Minh city, Vietnam [4]. Several studies were conducted to investigate treatment non-adherence among MMT patients in Vietnam [4, 10–14]. Their limitations included data collection in only one city or province, lack of information on social support, using simple questions for measuring social support, and/or direct interviews with patients to assess treatment non-adherence. In the context of expanding take-home methadone programs, researching treatment non-adherence is of paramount importance. Using a sample of 600 methadone maintenance patients from a previous study [15], this study was carried out to investigate the prevalence of treatment non-adherence and its associated factors among MMT patients living in both metropolitan and mountainous areas of Vietnam.

## Methods

### Study setting and sampling methods

One city (Hanoi) and two provinces (Dien Bien and Son La) were selected to collect data. The Hanoi capital can be representative of metropolitan areas with high population density (about 2,480 people/km^2^). Dien Bien and Son La provinces can be regarded as mountainous areas with low population density (66 and 91 people/km^2^, respectively). It is noted that during the data collection process, Dien Bien was one of three provinces selected to pilot take-home methadone programs in Vietnam (besides Lai Chau province and Hai Phong city).

A convenience sampling method was used to recruit eligible patients. Participants’ inclusion criteria included (1) being willing to participate in this research, (2) being 18 years old and above, and (3) receiving methadone medication at selected clinics with a treatment time of at least three months. At the beginning of data collection, the total number of methadone maintenance patients in the three investigated clinics was 1,013. The sample size to estimate a proportion was calculated using the formula: n = (Z/m)^2^.p.(1-p). With α = 0.05 (Z = 1.96), a margin of error of 5% (m = 0.05), and *p* = 0.382 [4], the minimum sample size was 363 patients. To increase the reproducibility and extrapolation of results, the research team strived to approach as many patients as possible. Among 623 patients approached from December 2021 to April 2022, 600 patients agreed to take part in this research (response rate: 96.3%).

### Procedures and measurements

Data were collected via two primary sources: 1) face-to-face interviews with patients and 2) medical records and books monitoring their treatment process. After taking methadone in MMT clinics, patients were invited to participate in this research. After signing the written informed consent, eligible patients were face-to-face interviewed by data collectors (Master’s and specialist students). The duration of each interview was approximately 15 min. Patients did not receive any remuneration or incentives for their participation.

The questionnaire used to interview patients consisted of two main parts. The first part comprised questions involving demographic characteristics of patients: (1) sex (male, female), (2) highest education level (illiterate, primary school, secondary school, high school, college, university or higher), (3) place of residence (Hanoi, Dien Bien, Son La), (4) occupation, (5) patient’s monthly income, (6) family’s monthly income, and (7) people living with the patient (family members). Participants were also asked whether or not they had missed taking methadone in the last month, reasons for non-adherence, and difficulties during their treatment process. The second part was 20 questions from the Medical Outcomes Study—Social Support Survey (MOS-SSS) questionnaire. A Vietnamese version of the MOS-SSS questionnaire was published, and its reliability and validity were confirmed [16]. The first question is about the total number of close friends/relatives whom the patient can feel at ease with and talk to about what is on his/her mind. Nineteen remaining questions measuring patient’s social support can be divided into four domains (emotional-information support: 8 questions, tangible support: 4 questions, positive social interaction: 3 questions, and affectionate support: 3 questions) and one additional question. Eligible answers for each question were none of the time, a little of the time, some of the time, most of the time, and all of the time (a five-point Likert rating scale). Patients’ average social support scores were calculated for these 19 questions (score range: 1–5). The higher scores indicated more social support [16, 17]. With the data of 600 patients, the good internal consistency of this questionnaire was demonstrated via Cronbach’s alpha (emotional-information support: 0.89, tangible support: 0.87, positive social interaction: 0.80, and affectionate support: 0.84, and the overall questionnaire: 0.95).

The treatment characteristics of patients were mainly collected from their medical records and books monitoring their treatment process. Collected information included (1) the patient’s year of birth, (2) initial drug use age, (3) duration of drug use (years), (4) the number of previous treatments, (5) time to start treatment in the current clinic, (6) comorbidity, (7) daily methadone dose, (8) urine opioid tests in the last three months, and (9) the number of missing methadone doses in the last three months. A patient was categorized as “complete adherence” if this person did not miss any methadone dose in the last three months. This criterion was taken from the treatment guideline of the Vietnam Ministry of Health and was also used in many previous studies [4, 13, 18]. Furthermore, to provide some additional information involving the group of non-adherent patients, the number of missing methadone doses in the last three months was reported via two subgroups: 1–8 missing doses (> 90% of methadone use days), and 9 missing doses or higher (≤ 90% of methadone use days) [7].

### Data analysis

The collected data were analyzed using R software version 4.3.2 (with the following packages: *table1, psych, ggplot2, gridExtra, epiDisplay, psfmi, Epi, FSA, BAS, car, ResourceSelection,* and *pROC*). Categorical variables (such as sex) were reported via numbers and percentages. Means with standard deviations (SD) and medians (interquartile range—IQR) were used to describe numeric variables (such as age). The normal distribution of a numeric variable was assessed using the *Shapiro–Wilk test* with a *p*-value > 0.05 indicating a normal distribution. Between two patient groups (adherence and non-adherence), differences in social support scores, the number of family members, and the number of close friends/relatives were analyzed via the *Wilcoxon rank-sum test.* The *Chi-squared test* and *Fisher’s exact test* were employed to assess the relationships between two categorical variables. Factors associated with treatment non-adherence were determined via univariate and multivariate logistic regression models. The *Bayesian Model Averaging* method was utilized to select variables in the final multivariate model. The goodness of fit of this multivariate model was assessed via the *Hosmer–Lemeshow test*, the area under the curve (AUC), and Nagelkerke's R-squared value. A *p*-value  < 0.05 was considered statistical significance.

## Results

### Patients’ demographic and treatment characteristics

Among 600 participants, most of them were males (98.3%) and those with the highest education level of secondary or high schools (76.0%). Nearly 90% of patients were aged 30 and above. A quarter of participants did not work, while freelancers (30.8%) and farmers (23.7%) were two common occupations among patients. On average, a patient earned about 119.07US$ per month, and this figure for the whole family was roughly 306.36US$. A patient lived with about one to four family members (87.2%) and had from two to five close friends/relatives (Table 1).

**Table 1:** Patients’ demographic characteristics and their treatment adherence (n = 600 patients)

Demographic characteristics		Number of patients (%)			
		All patients	Complete adherence (no missing doses)	Non-adherence	
				1 to 8 missing doses	9 missing doses or higher
Age	< 30	61 (10.2)	23 (37.7)	35 (57.4)	3 (4.9)
	30 to 39	175 (29.2)	85 (48.6)	81 (46.3)	9 (5.1)
	40 to 49	213 (35.5)	121 (56.8)	78 (36.6)	14 (6.6)
	50 and higher	151 (25.2)	97 (64.2)	42 (27.8)	12 (7.9)
Sex	Male	590 (98.3)	319 (54.1)	233 (39.5)	38 (6.4)
	Female	10 (1.7)	7 (70.0)	3 (30.0)	0 (0.0)
Place of residence (Province)	Dien Bien	192 (32.0)	126 (65.6)	53 (27.6)	13 (6.8)
	Hanoi	204 (34.0)	131 (64.2)	53 (26.0)	20 (9.8)
	Son La	204 (34.0)	69 (33.8)	130 (63.7)	5 (2.5)
Highest level of education	Secondary school and lower	335 (55.8)	160 (47.8)	151 (45.1)	24 (7.2)
	High school and higher	265 (44.2)	166 (62.6)	85 (32.1)	14 (5.3)
Living with somebody	Yes	564 (94.0)	306 (54.3)	227 (40.2)	31 (5.5)
	No	36 (6.0)	20 (55.6)	9 (25.0)	7 (19.4)
Number of family members living with the patient	Living alone	36 (6.0)	20 (55.6)	9 (25.0)	7 (19.4)
	1 – 2	298 (49.7)	166 (55.7)	112 (37.6)	20 (6.7)
	3 – 4	225 (37.5)	120 (53.3)	95 (42.2)	10 (4.4)
	5 and higher	41 (6.8)	20 (48.8)	20 (48.8)	1 (2.4)
Number of close friends/relatives	Noone	21 (3.5)	8 (38.1)	10 (47.6)	3 (14.3)
	1 to 2	248 (41.3)	137 (55.2)	95 (38.3)	16 (6.5)
	3 to 4	179 (29.8)	79 (44.1)	92 (51.4)	8 (4.5)
	5 and higher	152 (25.3)	102 (67.1)	39 (25.7)	11 (7.2)
Social support score	1 to < 2	53 (8.8)	21 (39.6)	25 (47.2)	7 (13.2)
	2 to < 3	101 (16.8)	52 (51.5)	39 (38.6)	10 (9.9)
	3 to < 4	255 (42.5)	117 (45.9)	124 (48.6)	14 (5.5)
	4 to 5	191 (31.8)	136 (71.2)	48 (25.1)	7 (3.7)
Occupation	Not working	154 (25.7)	94 (61.0)	43 (27.9)	17 (11.0)
	Farmer	142 (23.7)	48 (33.8)	87 (61.3)	7 (4.9)
	Freelancer	185 (30.8)	110 (59.5)	67 (36.2)	8 (4.3)
	Trader	41 (6.8)	26 (63.4)	13 (31.7)	2 (4.9)
	Other occupations	78 (13.0)	48 (61.5)	26 (33.3)	4 (5.1)
Patient’s monthly income (mVND)	< 3	323 (53.8)	149 (46.1)	148 (45.8)	26 (8.0)
	3 to < 6	198 (33.0)	122 (61.6)	68 (34.3)	8 (4.0)
	6 or higher	79 (13.2)	55 (69.6)	20 (25.3)	4 (5.1)
Family’s monthly income (mVND)	< 5	230 (38.3)	98 (42.6)	115 (50.0)	17 (7.4)
	5 to < 10	218 (36.3)	125 (57.3)	83 (38.1)	10 (4.6)
	10 and higher	152 (25.3)	103 (67.8)	38 (25.0)	11 (7.2)
Exchange rate: 1 million Vietnam dongs (mVND) = 42.373 US dollars					

The initial drug use age of most patients was under 30 years old (81.3%). Their average duration of drug use was 11.3 ± 7.93 years. On average, patients’ treatment time in the current treatment clinics was five years. Nearly half of the participants took 60–120 mg of methadone per day, while a third of them received a daily dose of less than 60 mg. Besides drug addiction, 321 patients (53.5%) had at least one comorbidity. The common comorbidities included hepatitis C (261 patients), Human Immunodeficiency Virus—HIV (63 patients), hepatitis B (61 patients), and tuberculosis (14 patients) (Table 2). In addition, patients’ difficulties during the treatment process included treatment costs (94 patients), treatment time (34 patients), and the long distance between their home and methadone clinics (two patients).

**Table 2:** Patients’ treatment characteristics and their treatment adherence

Treatment characteristics		Number of patients (%)			
		All patients	Complete adherence (no missing doses)	Non-adherence	
				1 to 8 missing doses	9 missing doses or higher
Initial drug use age	< 20	167 (27.8)	82 (49.1)	73 (43.7)	12 (7.2)
	20 to 29	321 (53.5)	182 (56.7)	121 (37.7)	18 (5.6)
	30 and higher	112 (18.7)	62 (55.4)	42 (37.5)	8 (7.1)
Duration of drug use (year)	< 5	115 (19.2)	58 (50.4)	51 (44.3)	6 (5.2)
	5 to < 10	186 (31.0)	109 (58.6)	66 (35.5)	11 (5.9)
	10 to < 15	133 (22.2)	68 (51.1)	52 (39.1)	13 (9.8)
	15 and higher	166 (27.7)	91 (54.8)	67 (40.4)	8 (4.8)
The number of previous treatment	No	124 (20.7)	53 (42.7)	65 (52.4)	6 (4.8)
	1 to 2	300 (50.0)	176 (58.7)	105 (35.0)	19 (6.3)
	3 or more	176 (29.3)	97 (55.1)	66 (37.5)	13 (7.4)
Treatment time (year)	< 3	185 (30.8)	56 (30.3)	121 (65.4)	8 (4.3)
	3 to 6	198 (33.0)	124 (62.6)	57 (28.8)	17 (8.6)
	> 6	217 (36.2)	146 (67.3)	58 (26.7)	13 (6.0)
Daily methadone dose (mg)	< 60	220 (36.7)	130 (59.1)	75 (34.1)	15 (6.8)
	60 to 120	298 (49.7)	150 (50.3)	129 (43.3)	19 (6.4)
	> 120	82 (13.7)	46 (56.1)	32 (39.0)	4 (4.9)
Comorbidity	No	279 (46.5)	146 (52.3)	118 (42.3)	15 (5.4)
	Yes	321 (53.5)	180 (56.1)	118 (36.8)	23 (7.2)
Urine opioid tests	Positive	68 (11.3)	23 (33.8)	41 (60.3)	4 (5.9)
	Negative	532 (88.7)	303 (57.0)	195 (36.7)	34 (6.4)

### Treatment non-adherence among methadone maintenance patients

Overall, the prevalence of complete adherence among 600 MMT patients was 54.3%. In other words, 274 patients missed at least one dose of methadone in the last three months, including 236 patients with 1–8 missing doses and 38 patients with nine missing doses or higher. The main reasons for missing doses included sickness/tiredness (36 patients), personal business/busy work (22 patients), traveling to places far from their clinics (11 patients), forgetting to take doses (9 patients), and heavy rain (2 patients).

The proportion of non-adherence in patients with high education levels (high school or higher, 37.4%) was significantly lower than that of those with low education levels (secondary school or lower, 52.2%) (*p* < 0.001). Among three provinces, treatment adherence among patients living in Son La was the worst (non-adherence: 66.2%) (*p* < 0.001). Regarding patients’ occupations, the prevalence of non-adherence among farmers was the highest (66.2%). The prevalence of treatment non-adherence was also high among patients with low monthly income (*p* < 0.001). A high proportion of non-adherence was found among patients with a treatment time of lower than three years (69.7%) in comparison with those having a treatment time of three to six years (37.4%) and more than six years (32.7%) (*p* < 0.001) (Tables 1, 2).

**Fig. 1:**
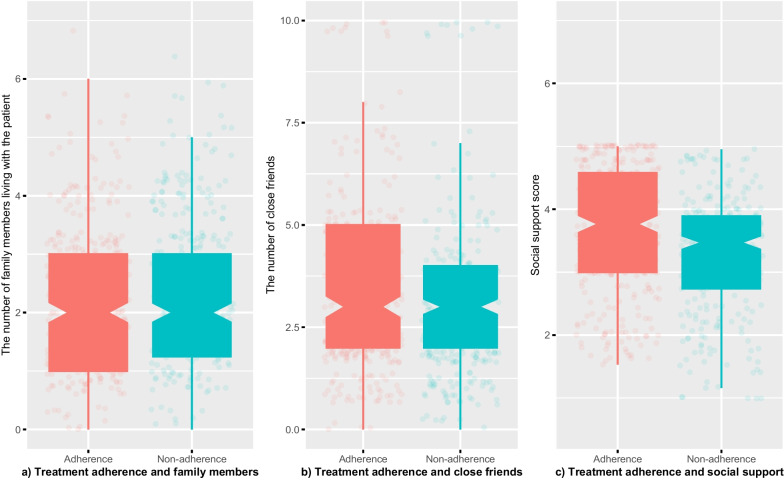
Treatment adherence and social support among methadone maintenance patients

Regarding social support, the average score of all patients was 3.49 ± 0.97 (median: 3.58, IQR: 2.95–4.16, range: 1–5). Being non-adherent among patients having low social support scores (from 1 to < 2) was 2.10 times more likely when compared with those with high scores (from 4 to 5) (*p* < 0.001). The average social support score of patients who completely adhered to MMT (3.68 ± 0.98) was significantly higher than that of those who did not (3.28 ± 0.92) (*p* < 0.001). The average number of close friends/relatives of the former (4.01) was also significantly higher than that of the latter (3.37) (*p* = 0.023). In addition, no significant difference was found when comparing the average number of family members living with adherents and that of non-adherents (*p* = 0.126) (Fig. 1).

### Factors associated with the treatment non-adherence of methadone maintenance patients

**Fig. 2:**
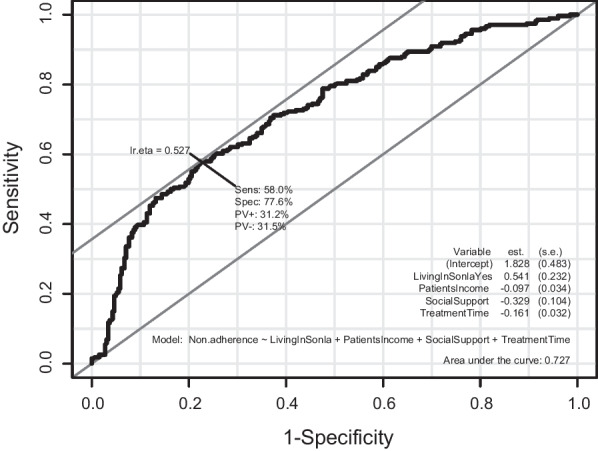
The receiver operating characteristic (ROC) curve analysis for the multivariate logistic regression model. Model: Non-adherence ~ Living in Son La (yes/no) + Social support (score) + Patient’s monthly income (million Vietnam dongs) + Treatment time (year). AUC (the area under the curve) = 0.7273 (95%CI 0.6869–0.7678)

As per the multivariate logistic regression model, each additional increase of one social support score was associated with a 28% decrease in the odds of being non-adherent (adjusted odds ratio (aOR) = 0.72, 95% confidence interval (95%CI) 0.59–0.88, *p* = 0.002). Patients living in Son La province were 1.72 times (95%CI 1.09–2.71) more likely to be non-adherent as compared to those in other areas (*p* = 0.020). For each one-unit increase in patient’s treatment time (a year) and monthly income (one million Vietnam dongs), the odds of non-adherence decreased by about 15% (aOR = 0.85, 95%CI 0.80–0.91, *p* < 0.001) and 9% (aOR = 0.91, 95%CI 0.85–0.97, *p* = 0.004), respectively (Table 3). The results from the Hosmer–Lemeshow goodness of fit test for the multivariate model showed that this model could adequately fit the data (χ^2^ = 11.019, df = 8, *p* = 0.201). The AUC of this model was 0.7273 (95%CI 0.6869–0.7678) (Fig. 2). The Nagelkerke's R-squared value was 0.200.

**Table 3:** Factors associated with treatment non-adherence among methadone maintenance patients

Independent variable	Univariate logistic regression		Multivariate logistic regression			
			With all independent variables		Using the BMA method to select variables (final model)	
	OR (95% CI)	*p*-value	aOR (95% CI)	*p*-value	aOR (95% CI)	*p*-value
1. Sex (ref: female)						
Male	1.98 (0.51–7.74)	0.325	1.56 (0.35–7.09)	0.562		
2. Age (years old)	0.96 (0.95–0.98)	< 0.001	0.99 (0.95–1.03)	0.634		
3. Place of residence (ref: Dien Bien)						
Hanoi	1.06 (0.70–1.61)	0.769	0.87 (0.47–1.59)	0.651		
Son La	3.74 (2.46–5.66)	< 0.001	1.29 (0.68–2.44)	0.439	1.72 (1.09–2.71)	0.020
4. Education level (ref: high school or higher)						
Secondary school or lower	1.83 (1.32–2.55)	< 0.001	1.53 (1.03–2.27)	0.035		
5. Occupation (ref: farmer)						
Not working	0.33 (0.20–0.52)	< 0.001	0.53 (0.26–1.07)	0.078		
Freelancer	0.35 (0.22–0.55)	< 0.001	0.71 (0.39–1.30)	0.272		
Trader	0.29 (0.14–0.61)	< 0.001	0.88 (0.35–2.19)	0.784		
Others	0.32 (0.18–0.57)	< 0.001	1.20 (0.56–2.58)	0.643		
6. Number of family members	1.09 (0.97–1.22)	0.134	1.15 (0.99–1.33)	0.063		
7. Patient’s income per month (mVND)	0.91 (0.85–0.96)	< 0.001	0.87 (0.78–0.98)	0.019	0.91 (0.85–0.97)	0.004
8. Family’s income per month (mVND)	0.94 (0.91–0.97)	< 0.001	1.00 (0.95–1.05)	0.909		
9. Number of close friends/relatives	0.94 (0.90–0.99)	0.026	1.03 (0.96–1.09)	0.414		
10. Social support score	0.65 (0.54–0.77)	< 0.001	0.65 (0.51–0.83)	< 0.001	0.72 (0.59–0.88)	0.002
11. Initial drug use age	0.99 (0.97–1.01)	0.334	1.01 (0.97–1.05)	0.692		
12. Duration of drug use (year)	1.00 (0.98–1.02)	0.999	0.98 (0.94–1.02)	0.310		
13. The number of previous treatment	0.94 (0.86–1.04)	0.246	1.18 (1.05–1.34)	0.008		
14. Daily methadone dose (mg)	1.00 (1.00–1.00)	0.333	1.00 (1.00–1.00)	0.763		
15. Treatment time (year)	0.83 (0.79–0.87)	< 0.001	0.86 (0.79–0.92)	< 0.001	0.85 (0.80–0.91)	< 0.001
16. Urine opioid test (ref: Negative)						
Positive	2.59 (1.52–4.40)	< 0.001	2.05 (1.09–3.87)	0.026		
17. Comorbidity (ref: No)						
Yes	0.86 (0.62–1.19)	0.358	0.93 (0.62–1.40)	0.737		

Exchange rate: 1 million Vietnam dongs (mVND) = 42.373 US dollars

OR: odds ratio, aOR: adjusted odds ratio, 95% CI: 95% confidence interval, ref: reference. BMA: Bayesian Model Averaging

Besides the four above factors, according to the univariate logistic regression models, patient’s treatment non-adherence could be associated with their age, education level, occupation, family income per month, the number of close friends/relatives, and opioid relapse. Patients with an education level of high school or higher experienced a reduction of 45% in the odds of non-adherence in comparison with those with lower education levels (*p* < 0.001). The odds of non-adherence slightly declined by 4% for each increase of one year in patient’s age (OR = 0.96, 95%CI 0.95–0.98, *p* < 0.001) and 6% for each increase of one close friend/relative (OR = 0.94, 95%CI 0.90–0.99, *p* = 0.026). Patients who relapsed into opioid use in the last three months were 2.59 times (95%CI 1.52–4.40) more likely to be non-adherent as compared to those not using opioids (*p* < 0.001). In addition, the patient’s sex, the number of family members, initial drug use age, duration of drug use, the number of previous treatments, daily methadone dose, and comorbidity had no significant associations with treatment non-adherence among MMT patients (*p* > 0.05) (Table 3).

## Discussion

This study was conducted in multiple geographical areas in Vietnam to evaluate treatment non-adherence and its associated factors among MMT patients. In comparison with previous studies, using a highly reliable and validated instrument to measure social support and assessing treatment non-adherence based on medical records and books monitoring patients’ treatment process were the two main strengths of our study. This research indicates a high prevalence of treatment non-adherence among MMT patients in Vietnam. Notable risk factors of non-adherence included younger age, living in mountainous and rural areas, being a farmer, lower education level, lower economic condition, shorter treatment time, lower social support, not having many close friends/relatives, and opioid relapse. The authorities and stakeholders can focus on these findings to have practical solutions to promote treatment adherence among MMT patients in Vietnam.

The prevalence of treatment non-adherence among MMT patients in this study was 45.7%, far lower than the results of previous studies in China (Yunnan: 73.4%, Guangzhou: 62%, Xi’an: 52.2%) [6–8]. This figure was 47.5% and 27.9% in Canada and Nepal, respectively [9, 19]. In comparison with previous studies in Vietnam, our figure was lower than the findings of studies in Tuyen Quang (65.6%) [13] and Nam Dinh (56.7%) [10] but higher than the result in Ho Chi Minh city (38.2%) [4]. The prevalence of treatment non-adherence among MMT patients varies across countries and areas. Several possible reasons are the heterogeneity in the duration (one month, three months, or a year) and sources used to assess treatment adherence (direct interviews with patients or medical records).

People living in Son La province were related to a higher likelihood of non-adherence. Son La is a mountainous province bordering Laos. By virtue of geographical barriers, it is difficult for the authorities and police officers to control and impede illegitimate activities such as growing poppy plants and trading drugs, especially in border areas. Rough terrain, low population density, low education, and penury can be factors hindering the patients’ access to methadone clinics in this province. Dien Bien is also a mountainous province in Vietnam. However, the non-adherence rate among MMT patients in this province was lower than that of Son La. A possible rationale is that Dien Bien has been one of three provinces selected to pilot take-home methadone programs since April 2021. If a patient wants to take methadone at home, this person must evince strict adherence to MMT for a long time. In 2023, these programs have been expanded and applied nationwide in Vietnam. This can generate motivation and influence adherence behaviors among Vietnamese MMT patients.

Among other demographic characteristics, patients with higher economic conditions and higher education were also more likely to be adherent. Patients with high education levels and economic conditions may have much knowledge about the pernicious effects of non-compliance and the benefits of MMT. In China and the United States, patients with low income per month were also less likely to maintain MMT [20], while those with low education levels had a greater likelihood of non-adherence and dropout [21, 22]. Besides, many farmers did not adhere to the MMT in this study. Farmers had lower education levels (secondary schools or lower: 76.1%) and monthly income (80.51US$) in comparison with other occupation groups (secondary schools or lower: 49.6%, monthly income: 131.13US$). The inability to pay treatment costs can be a rationale behind patients’ treatment non-adherence and opioid treatment program discharge [23].

Social support can be a significant factor associated with treatment adherence and retention among MMT patients. Social support also plays a crucial role in reducing stress and depression, improving health-related quality of life, lowering the risk of opioid relapse, and helping MMT patients overcome stigma and discrimination [24, 25]. Longer retention was also found among patients receiving better support from their families, friends, health workers, and public security departments [19, 20, 26, 27]. In China, patients with poorer perceived social support were 1.25 times (95%CI 1.04–1.51) more likely to terminate MMT in comparison with those with good perceived social support [28]. In this study, the decrease by 28% and 6% in the odds of non-adherence for each one-unit increase in social support score and the number of close friends/relatives may demonstrate the role of social support in the treatment process of MMT patients. In addition, the average social support score of patients in Hanoi (a metropolitan area) was significantly higher than that of those living in Dien Bien and Son La (mountainous areas). The lack of social support among patients can be another reason for the low adherence rate in mountainous areas. During the treatment, MMT patients have to face many difficulties and challenges [29–33]. In the context of the withdrawal of international funding sponsors and the restriction of the national budget, patients’ families, friends, and society can join hands with the government to support MMT patients in their lives and treatment process.

Regarding factors involving patients’ treatment process, a higher level of adherence to MMT was witnessed among Vietnamese patients who were treated for a longer time. There was a positive correlation between patients’ age and treatment time in the current clinics. Similar to our finding, older age was also a positive factor associated with treatment adherence and retention in previous studies [10, 20, 34]. Younger patients can be more easily influenced by their peer drug users [7, 26]. Those with higher age and longer duration of treatment may know the exorbitant prices and detrimental effects of drug use. They were also aware of the many benefits of MMT, felt regrets, and wanted to start a new life [35, 36]. We also found a significant relationship between patients’ current drug use (opioid relapse) and treatment non-adherence, in line with the results of many previous studies [4, 7, 10]. In several previous studies, methadone doses can affect patients’ retention and attendance rates [37]. However, our findings showed that the association between this factor and treatment non-adherence was insignificant.

 This study has several following limitations. First and foremost, by reason of only collecting data at one point in time, causal associations between treatment non-adherence and associated factors cannot be determined in this cross-sectional study. Second, the generalizability of findings may be limited due to using a convenience sampling method to recruit participants. Third, some factors, such as patients’ satisfaction with the quality of MMT services and the time taken to go to methadone clinics, were not included (although in this study, when patients were asked about their difficulties during the treatment process, only two patients mentioned the distance between their houses and clinics). By reason of not including specific organizational MMT variables, we cannot examine the relationship between treatment non-adherence and factors associated with the treatment setting and/or MMT regulation policies. In addition, some biases can occur in secondary data analysis studies, such as researcher bias and selective reporting bias. Last but not least, the multivariate logistic regression model may not be widely used to predict treatment non-adherence among MMT patients by virtue of the moderate level of AUC and Nagelkerke's R-squared values.

## Conclusions

A high rate of treatment non-adherence was found among MMT patients in northern Vietnam. As per the multivariate logistic regression model, factors significantly associated with patients’ non-adherence included their place of residence, patients’ monthly income, social support, and treatment time. According to univariate analyses, treatment non-adherence can be associated with patient age, occupation, education level, family’s monthly income, the number of close friends/relatives, and opioid relapse.

## Data Availability

The datasets used and/or analysed during the current study are available from the corresponding author on reasonable request.

## Abbreviations

95%CI: 95% Confidence interval
aOR: adjusted odds ratio
AUC: area under the curve
HIV: human immunodeficiency virus
IQR: interquartile range
MOS-SSS: medical outcomes study—social support survey
MMT: methadone maintenance treatment
ref: reference
SD: standard deviation
VND: Vietnam dong
WHO: World Health Organization
